# Microglial Scavenger Receptors and Their Roles in the Pathogenesis of Alzheimer's Disease

**DOI:** 10.1155/2012/489456

**Published:** 2012-05-15

**Authors:** Kim Wilkinson, Joseph El Khoury

**Affiliations:** Center for Immunology and Inflammatory Diseases, Massachusetts General Hospital, Harvard Medical School, Charlestown, MA, 02129, USA

## Abstract

Alzheimer's disease (AD) is increasing in prevalence with the aging population. Deposition of amyloid-**β** (A**β**) in the brain of AD patients is a hallmark of the disease and is associated with increased microglial numbers and activation state. The interaction of microglia with A**β** appears to play a dichotomous role in AD pathogenesis. On one hand, microglia can phagocytose and clear A**β**, but binding of microglia to A**β** also increases their ability to produce inflammatory cytokines, chemokines, and neurotoxic reactive oxygen species (ROS). Scavenger receptors, a group of evolutionally conserved proteins expressed on the surface of microglia act as receptors for A**β**. Of particular interest are SCARA-1 (scavenger receptor A-1), CD36, and RAGE (receptor for advanced glycation end products). SCARA-1 appears to be involved in the clearance of A**β**, while CD36 and RAGE are involved in activation of microglia by A**β**. In this review, we discuss the roles of various scavenger receptors in the interaction of microglia with A**β** and propose that these receptors play complementary, nonredundant functions in the development of AD pathology. We also discuss potential therapeutic applications for these receptors in AD.

## 1. Microglia and Alzheimer's Disease Pathology

Alzheimer's disease (AD) is a devastating neurological condition characterized by increasing memory loss, inability to perform daily tasks, and eventually dementia. This fatal condition currently has no cure, and by 2050, 13 million people in the United States are projected to be affected by the disease [1]. AD brains show deposition of the protein amyloid-*β* (A*β*) in senile plaques [2], this protein is produced by cleavage of APP (amyloid precursor protein), by the enzymes *β*-secretase and *γ*-secretase [3]. A*β* accumulates in soluble form and also undergoes conformational changes to become fibrillar, microglia interact with both soluble and fibrillar forms of A*β* [4–6].

In addition to A*β* accumulation during the development of AD, tau protein also accumulates in neurofibrillary tangles (NFT) in cell bodies of neurons [7] and apical dendrites [8]. Tau is a microtubule-associated protein that segregates into axons and stabilizes their microtubules. In AD, tau dissociates from the microtubules and begins to accumulate in the somatodendritic compartment of the axon, a process which is not fully understood [9]. Tau contains a high number of phosphorylation sites and upon phosphorylation dissociates from the microtubules, as observed in AD [10]. Tau protein then undergoes conformational changes which form fibrils [11]. NFT could possibly activate microglia which may prove deleterious for the surrounding neurons and contribute to disease progression, however, little is known about the NFT mechanism of action [12].

Microglia are the major phagocytic cell of the brain and become activated upon encountering A*β* [6] and can release chemokines and cytokines into their environment [13]. Microglia are believed to initially clear A*β* deposits, but as the disease progresses, they produce proinflammatory cytokines, chemokines, and reactive oxygen species (ROS) and lose their ability to clear A*β* [14], and despite increased numbers of microglia in the AD brain, the plaques continue to increase in size and number [15]. This inflammatory environment ultimately becomes toxic to the surrounding neurons, resulting in neuronal degeneration and disease progression [16].

Scavenger receptors bind many ligands with high affinity, including A*β* [4, 17], and have been shown to be expressed on microglia surrounding A*β* plaques in the brain [18]. This review will focus on the role of scavenger receptors in AD development and discuss efforts to examine scavenger receptors as targets for therapy.

## 2. What Are Scavenger Receptors (SRs)?

Several families of pattern recognition receptors (PRR) have been identified including the well-defined and extensively studied Toll-like and Nod-like receptors (TLR and NLRs). In addition to these proteins, the scavenger receptor (SR) family represents another major class of PRR. SRs were first described in 1979 by Brown and Goldstein as macrophage receptors that mediate endocytosis of modified low-density lipoprotein (LDL) leading to foam cell formation and were shown to play a role in the pathogenesis of atherosclerosis [19, 20]. Since then, the definition of SRs has been broadened: SRs are defined as a family of molecules that share the ability to bind polyanionic ligands. This simple definition belies the importance of SRs as PRRs-SRs are archetypal multifunctional receptors, often able to bind ligands of both pathogen and self-origin. These receptors are teleologically ancient pathogen receptors, emphasizing their important role in host defense. However, while the critical roles SRs play in atherosclerosis and host defense against a variety of pathogens is well characterized, the exact role of these receptors in the pathogenesis of neurodegenerative disorders including AD is not clearly understood.

## 3. Classification of SRs

**Figure 1:**
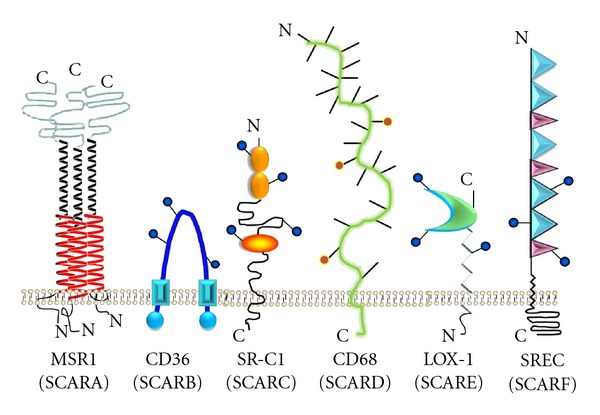
Structural diversity of the scavenger receptors. SCARA have a collagen-like domain believed to be their ligand-binding domain. CD68 has a mucin-like domain; LOX-1(oxidized-low density lipoprotein (lectin-like) receptor-1) (SCARE) has a C-type lectin domain and binds oxidized LDL. SCARF (scavenger receptor class F) has multiple extracellular EGF-like repeats.

SRs are structurally unrelated membrane receptors that are highly expressed by phagocytes such as macrophages, dendritic cells, and microglia, and also found on selected endothelial cells (Figure 1). To date, the SRs family has been classified into 6 classes but additional members of this family, like CD163, RAGE, and SR-PSOX (scavenger receptor that binds phosphatidylserine and oxidized lipoprotein) remain unclassified. SRs are defined by their common ability to bind polyanionic ligands with high affinity and have broad specificity. SRs cooperate with the other innate immune PRRs like the TLRs to define pathogen-specific responses [21]. However, unlike the TLRs and NLRs, SRs facilitate ligand uptake by phagocytosis and endocytosis [22].

## 4. Class A Scavenger Receptors (SCARA)

SCARA are required for host defense against several bacterial and viral pathogens: SCARA-1 is an arrangement of three coiled extracellular regions with cysteine-rich domains connected to the plasma membrane by a long fibrous stalk composed of an alpha-helical coiled coil and a collagen rich triple helix which is believed to bind ligands through the collagen-like domain [23–25] (Figure 1). SCARA-1 exemplify this family of multiligand receptors, in addition to binding to modified lipoproteins, lipopolysaccharide (LPS), [26] and lipoteichoic acid (LTA) [27, 28], we and others have shown that SCARA-1/2 also bind fibrillar *β*-amyloid and advanced glycosylation end products (AGEs) [4, 25]. SCARA-1/2 mediate phagocytosis of infectious organisms like *Staphylococcus aureus *[28] and *Neisseria meningitides *[29] and also mediate clearance of apoptotic thymocytes [30]. Because of their broad ligand specificity, SCARA-1/2 contribute to resistance to gram-positive and gram-negative microbial infections and some viral infections *in vivo*, and SCARA-1/2 null mice succumb to *S. aureus*, and *Herpes simplex* infections faster than normal mice [28, 31]. In addition, SCARA-1/2 null mice also demonstrate increased susceptibility to LPS-induced shock, [32] suggesting a potential role in regulating the endotoxin response.

### 4.1. SCARA Role in Alzheimer's Disease

Murine microglia bind and phagocytose A*β* via SCARA1. Studies using a transgenic mouse model of AD expressing the human form of A*β*, and developing A*β* plaques in the brain over time [33] showed an increased level of SCARA-1 on microglia around A*β* plaques [34]. Microglia isolated from human brains were also able to bind to and ingest A*β* via SCARA-1 [35]. Additional evidence that SCARA-1 is an important phagocytic receptor for A*β* comes from studies using microglia from SCARA-1 knock-out mice. SCARA-1 knock-out microglia isolated from these animals showed a 60% decrease in the ability to take up amyloid-*β* compared with wild-type cells [36]. These results suggest that in addition to SCARA-1, other receptors may be involved in clearance of A*β* by microglia. Since SCARA-1 is involved in clearance of A*β*, from a therapeutic standpoint, it may be beneficial to upregulate expression of this receptor on microglia thereby increasing the ability of these cells to clear A*β*.

Another SCARA that has been shown to interact with A*β* is the macrophage scavenger receptor with collagenous structure (MARCO). MARCO is a SCARA family member but is encoded by a distinct gene from SCARA-1/2. Importantly, MARCO and SCARA1/2 have both common and distinct ligands [37, 38]. MARCO is expressed on cultured neonatal rat microglia and is also a receptor for A*β* [39]. It is not known if MARCO is expressed in microglia in the AD brain. By coimmunoprecipitation, MARCO has been shown to form a complex with formyl peptide-receptor-like 1 (FPRL1) upon encountering A*β*. Intracellular signaling via ERK 1/2 and inhibition of cAMP is then initiated through FPRL1 which may play a part in decreasing the inflammatory response mediated through MARCO in microglia [40].

## 5. Class B Scavenger Receptors (SCARB)

SCARB are also important in the innate host response to bacterial and fungal pathogens. The B class receptors are characterized by the presence of membrane-spanning N and C termini and a large extracellular loop [41] (Figure 1). CD36 (SCARB-2), the first classified SCARB was initially identified as a receptor for thrombospondin [42] and for malaria parasitized erythrocytes [43]. Endemann and colleagues subsequently identified CD36 as the “second” modified lipoprotein receptor [44], the first receptor being SCARA-1/2. By virtue of their ability to bind HDL and act as fatty acid transporters, CD36 and SCARB-1 play major roles in cholesterol metabolism [45–47]. CD36 also binds and internalizes *S. aureus* and is required for protection against this pathogen [21]. Recently, we have uncovered a novel role for CD36 as a receptor for *β*-glucan [48]. We also found that CD36 and its *C. elegans* ortholog play a major role in host defense to opportunistic infections with pathogenic yeast such as *Candida albicans and Cryptococcus neoformans*. Interestingly, SCARB-1 act as a coreceptor for the hepatitis C virus and may play a role in viral pathogenesis of hepatitis C [49, 50]. In addition to binding to microbial ligands, CD36 binds several modified “self” antigens including AGE-modified proteins involved in the pathogenesis of vascular complications of diabetes [51].

### 5.1. SCARB Role in Alzheimer's Disease

Scavenger receptor B1 (SCARB-1) is a receptor for A*β* on microglia. A study by Husemann et al. [52] proposed that SCARB-1 is developmentally regulated and was not found on microglia in adult human brain and was only expressed on astrocytes. In a mouse model of AD, a reduction in SCARB-1 protein expression increased A*β* plaque deposition had no effect on microglial accumulation around A*β* plaques and in fact worsened cognitive defects in learning and memory [53].

CD36/SCARB-2 is also expressed on microglia and a receptor for amyloid-*β* [54]. Intracellular signaling through CD36 is activated upon A*β* binding and activates microglia to produce cytokines and chemokines that induce microglial migration [55]. CD36 knock-out microglia have less A*β*-mediated activation and reduced chemokines and cytokine production compared with wild-type cells. Injection of A*β* into CD36 knock-out mouse brains induced less accumulation of microglia compared with wild-type brains, demonstrating an important role for CD36 in the inflammatory response to A*β* [54]. Microglial CD36 signals upon A*β* engagement via the Src family members Fyn and Lyn and by activation of mitogen-activated protein kinase (MAPK) and subsequent chemokine and ROS production [56].

Further investigation of the signaling properties of CD36 has recently been shown to be intimately involved with two toll-like receptors TLR-4 and TLR-6. Upon A*β* engagement, CD36 forms a heterodimeric complex with TLR-4 and TLR-6 on microglia resulting in ROS production, and an increase in IL-*β* mRNA, indicative of inflammasome activation [5].

From a therapeutic standpoint, it may be advantageous to inhibit the ability of CD36 to signal when bound to A*β*, and thus prevent the release of chemokines and ROS by microglia that are deleterious to the surrounding neurons. We recently undertook a high-content screen to identify small molecule inhibitors of CD36. Screening of an FDA-approved compound library discovered ursolic acid to be an inhibitor of CD36 binding to A*β*. In addition, ursolic acid also inhibited A*β*-mediated ROS production in microglia without effecting microglial ability to phagocytose A*β* [57].

## 6. Class C Scavenger Receptors (SCARC)

SCARC-1 was identified in *Drosophila *but no mammalian ortholog for this receptor has yet been discovered. SCARC-1 is involved in phagocytosis of Gram-negative and Gram-positive bacteria but not yeast [58, 59]. Intriguingly, SCARC-1 also mediates uptake of dsRNA [59]. Since no mammalian ortholog for SCARC has been identified, it remains to be determined if this class of SR plays any role(s) in mammalian physiology or disease pathogenesis.

## 7. Class D Scavenger Receptors (SCARD)

The class D SRs are characterized by the presence of a mucin-like extracellular domain (Figure 1). The best characterized SCARD is CD68 (also known as macrosialin), which is expressed by macrophages, dendritic cells microglia, and osteoclasts [60, 61] For this reason, CD68 has been used for many years as a histological marker for these cells. It is found mainly intracellularly in late endosomes but cell surface expression increases following activation [62]. CD68/macrosialin plays a minor role in the binding and uptake of oxidized lipoproteins and apoptotic cells by macrophages [63].

### 7.1. Role of SCARD in Alzheimer's Disease

Studies investigating a role of SCARD in AD have been mostly descriptive. Immunizing human subjects with AD with A*β* 1–42 caused a reduction in plaques and an upregulation of CD68 expressed on microglia [64]. However in two longer surviving subjects, even though A*β* plaques were cleared, the expression of CD68 was found to be lower than in nonimmunized AD brains [65]. No studies to date investigated whether this class of SRs is mechanistically involved in CNS disorders including AD.

## 8. Class E Scavenger Receptors (SCARE)

Lectin-like oxidized LDL receptor (LOX-1) was the first scavenger receptor with a C-type lectin-like domain (Figure 1) to be identified. LOX-1 was initially cloned from endothelial cells and appears to play a role in atherosclerosis [66]. LOX-1 is expressed on freshly isolated human monocytes [67], LOX-1 decreases with monocyte differentiation into macrophages. LOX-1 binds oxidized LDL [66] and has also been implicated in the transport of *β*-amyloid across the blood brain barrier [68, 69]. In addition, LOX-1 binds gram positive and gram negative bacteria, although its exact role in the innate immune response to these pathogens is unknown [70]. It is not known if LOX-1 is expressed on microglia.

## 9. Class F Scavenger Receptors-SCARF

SCARFs are characterized by the presence of multiple extracellular epidermal growth factor-like repeats (Figure 1). The first SCARF was identified as an endothelial receptor for modified LDL, termed SREC [71]. In addition to binding modified LDL, SCARFs are receptors for heat shock proteins [72] and calreticulin which is involved in trafficking associated peptides into the major histocompatibility complex class I cross-presentation pathway of antigen-presenting cells [73]. We found that SCARF-1 is expressed on macrophages, and that SCARF-1 plays an important role in binding of the pathogenic yeasts *Candida albicans *and* Cryptococcus neoformans. *SCARF appears to be conserved through evolution. CED-1, a *C*. *elegans* ortholog of SCARF also appears to play a major role in the innate worm immune response to pathogenic yeast, and CED-1-deficient mutant worms have a dramatic increase in their susceptibility to these infections [48]. Interestingly, CED-1 also binds to cell corpses [74]. Similarly, related families of molecules have been identified in *Drosophila melanogaster* where they also contribute to host defense [75].

### 9.1. Role of SCARF in Alzheimer's Disease

It is not known if SCARF1 is expressed in the brain and/or involved in AD pathogenesis. MEGF10 (multiple EGF-like domains-10), also a member of the SCARF family, has recently been shown to be a receptor for A*β* [76]. MEGF10 is a type 1 transmembrane protein containing 17 EGF-like domains in the extracellular portion (Figure 1) [77]. MEGF10 is expressed in the brain of a transgenic mouse model of AD in the hippocampus and cortex region of the brain where A*β* plaques are also found, but it is unknown whether MEGF10 is expressed on microglia [76].

## 10. Unclassified Scavenger Receptors

### 10.1. RAGE

 RAGE (receptor for advanced glycation end products) is a member of the immunoglobulin superfamily of receptors [78] expressed on endothelial cells [79] and microglia which is capable of binding many ligands including A*β* [80], AGEs (advanced glycation end products) and S100 protein [81]. Ligand binding to RAGE induces many intracellular signaling pathways such as Ras-extracellular signal-regulated kinase 1/2 (ERK1/2) [82], Cdc42/Race [83] stress-activated protein kinase/c-Jun-NH2-terminal kinase (SAPK/JNK), and p38 mitogen-activated protein (MAP) kinase pathways [84] that activate transcription factors, for example, NF*κ*B [85], cAMP response element-binding (CREB) protein [82], or (STAT3), a member of the signal transducers and activators of transcription family [86]. RAGE activation through ligand binding induces a positive signaling feedback loop causing sustained activation of NF*κ*B and a chronic state of inflammation [87].

#### 10.1.1. Role of RAGE in Alzheimer's Disease

RAGE expressed on endothelial cells has previously been shown to play a role transporting A*β* into the brain [88], and also increasing the diapedesis of monocytes across the blood- brain barrier through RAGE-mediated signaling [89].

RAGE is expressed in higher levels on neurons and vasculature in AD brains compared with undiseased brains [90]. Soluble A*β* bound to RAGE induces microglial activation and chemotaxis along a concentration gradient, which may lead to microglial accumulation around A*β* plaques. [80].

In a double-transgenic mouse model of AD (both expressing mutated human APP and increased levels of RAGE), A*β* deposition, and microglial activation through NF*κ*B were observed, providing evidence that RAGE also functions as a signaling receptor [91]. Studies using transgenic mice expressing human A*β* and a dominant negative form of RAGE showed microglial RAGE to be an essential signaling receptor, signaling through p38 MAPK and JNK, which leads to synaptic dysfunction through JNK-mediated IL-*β* release [92].

However, more recent evidence shows that transgenic mice expressing both the Swedish and Arctic forms of human APP, and deficient in RAGE have a decrease in A*β* deposition and an increase in insulin degrading enzyme (IDE), an enzyme known to cleave A*β* when compared to mice expressing RAGE [93]. Such decrease in A*β* was found at 6 months of age. No improvement in cognitive function or difference in microglial recruitment to plaques was seen in 12 months old mice. This suggests RAGE may not be essential for microglial recruitment but could be involved in A*β* processing in the early disease state [94].

### 10.2. CD163

CD163 contains nine scavenger receptor cysteine-rich (SRCR) domains, an ancient and highly conserved protein motif, belonging to the SRCR superfamily. CD163 is expressed on mature tissue macrophages and has previously been shown to be involved in the clearance of hemoglobin-haptoglobin from the circulation [95]. Engagement of macrophage CD163-induced nitric oxide, IL-1*β*, and TNF*α* production, suggesting CD163 may be involved in activation of macrophages at sites of inflammation [96]. CD163 is also involved in host defense against both gram-positive and gram-negative bacteria, acting as an immune sensor and mediator of inflammation [97]. In the brains of patients with HIV-associated dementia, CD163 was found to be expressed on microglia [98], and on perivascular macrophages near the blood-brain barrier, [99] and CD163 is considered a marker for perivascular macrophages. However it is unknown whether CD163 is involved in the pathogenesis of AD.

### 10.3. SR-PSOX

Scavenger receptor that binds phosphatidylserine and oxidized lipids (SR-PSOX), also known as CXCL16, is a class H SR that can be both a membrane-bound and soluble protein. SR-PSOX is expressed on dendritic cells, and on monocyte-derived macrophages surrounding atherosclerotic plaques but not on smooth muscle cells or endothelial cells [100]. The membrane-bound form is composed of type I transmembrane glycoprotein, consisting of CXC chemokine, mucin stalk, transmembrane, and cytoplasmic domains [101]. Treatment of human peripheral blood mononuclear cells (PBMCs) with IFN*γ* induced increased uptake of oxLDL and increased levels of SR-PSOX [102]. The soluble form of SR-PSOX has been shown to ligate the T-cell receptor CXCR6 and attracts IFN*γ*-producing T cells to lymph nodes [103]. It is not known if this receptor binds A*β* or whether it plays a role in AD pathogenesis.

## 11. SR and Innate Immune Signaling

A common emerging theme is that SRs are not only innate immune recognition and phagocytic receptors but also act as critical regulators of inflammatory signaling and function as sensors for the innate immune response. The mechanism(s) by which SRs regulate the outcome of ligand engagement is currently unknown.

Two potential and not mutually exclusive possibilities exist. First, SRs may facilitate ligand delivery to other PRRs, particularly to the TLRs, which have not been shown to be sufficient for cellular binding to their targets. Pertinent to this, recent work has demonstrated that many TLRs do not interact with their ligands on the cell surface but within intracellular compartments such as endosomes or phagosomes. However, the mechanisms that deliver TLR ligands to the appropriate compartments are poorly defined. One possibility is that ligand delivery is facilitated by coreceptors such as Dectins [104–107] and SRs. The second scenario is that signals triggered directly from the SRs combine with those from other PRRs to define the ligand-specific response.

Such dichotomous cooperative role of SRs with other PRRs is nicely illustrated by CD36. We have observed that CD36 knockout macrophages and microglia show a decreased response to bacterial and self-ligands including A*β* but expression of CD36 is not by itself sufficient to initiate such responses. CD36 requires the presence of TLRs to mediate the responses to its ligands [5]. This reflects two roles for CD36 which contributes to response by both binding the ligand and also by synergistic and cooperative signaling with other PRRS such as TLRs. This cooperation may define the specificity of the response to a particular ligand. Indeed, we found that CD36/TLR2/6 respond to pathogens [108] whereas CD36/TLR4/6 respond to endogenous ligands such as *β*-amyloid and oxLDL. These roles for CD36 have been mostly described *in vitro*. It remains to be determined if such sophisticated ligand-receptor specificity of interaction also occurs *in vivo* and whether it affects diseases processes such as AD.

## 12. Diverse Roles of SRs in AD

**Figure 2:**
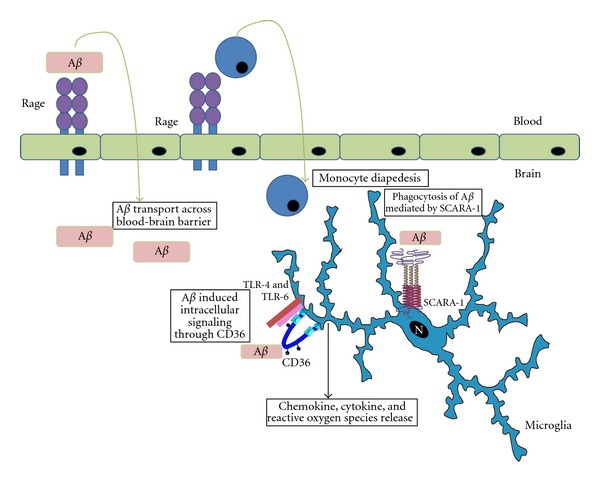
Diverse functions of SRs in development of Alzheimer's disease. RAGE facilitates transport of monocytes and A*β* across the blood-brain-barrier, whereas SCARA-1 mediates internalization of A*β* by microglia. CD36 and TLR-4 and TLR-6 ligation with A*β* induces intracellular signaling and release of ROS, chemokines and cytokines.

Promising evidence has shown the diversity of SRs with regards to their functions during the development of AD. SCARA-1 and CD36 play complementary nonredundant roles in the interactions of microglia with A*β*. SCARA-1-A*β* interactions are beneficial and promote phagocytosis and clearance of A*β*, whereas CD36-A*β* interactions are harmful and together with TLR-4 and TLR-6 lead to production of neurotoxins and proinflammatory molecules. This is reminiscent of the role CD36 plays in the interaction between macrophages and oxidized low-density lipoproteins in atherosclerosis. Indeed, we have shown in the past that while SCARA-1 mediates adhesion to oxidized LDL coated surfaces, CD36 mediates macrophage activation by oxidized LDL to produce reactive oxygen species [109]. Such differential roles of SCARA-1 and CD36 may have therapeutic implications for AD. Indeed, because of the role of SCARA-1 in A*β* clearance, drugs that upregulate SCARA-1 expression or functions may be helpful for treatment of AD. In contrast, drugs that block CD36 interactions with A*β* or reduce its surface expression may be helpful to stop or delay progression of AD. Similarly, since RAGE expressed on endothelium facilitates the transport of circulating A*β* from the blood across the blood-brain barrier into the brain, compounds that block A*β*-RAGE interactions may be beneficial. In addition to SCARA-1, CD36 and RAGE, and other SRs such as MARCO, LOX-1, and MEGF-10 are also emerging as receptors that bind A*β* and/or may have a role in the pathogenesis of disease (Figure 2). Dissecting the complex roles of various scavenger receptors in microglia-A*β* interactions, is therefore, important to understand the role of microglia in this disease and has therapeutic implications for treatment of this devastating disorder.
